# Editorial: Frontier research on artificial intelligence and radiomics in neurodegenerative diseases

**DOI:** 10.3389/fneur.2025.1705160

**Published:** 2025-10-14

**Authors:** Shi-Nan Wu, Yu-Qi Jiang, Qi Zhang, Wencai Liu

**Affiliations:** ^1^School of Medicine, Xiamen University, Xiamen, Fujian, China; ^2^Department of Genetic, School of Medicine, Yale University, New Haven, CT, United States; ^3^Department of Orthopedics, Shanghai Sixth People's Hospital Affiliated to Shanghai Jiao Tong University School of Medicine, Shanghai, China

**Keywords:** artificial intelligence, neurodegenerative diseases, radiomics, machine learning, Parkinson's disease

Neurodegenerative diseases, such as Alzheimer's disease (AD), Parkinson's disease (PD), and amyotrophic lateral sclerosis, impose an ever-growing burden on patients, families, and healthcare systems worldwide (1). Despite remarkable advances in neuroscience, early diagnosis and effective therapeutic monitoring remain challenging. In recent years, artificial intelligence (AI) and radiomics have emerged as powerful tools to transform the landscape of neurodegenerative disease research (2). AI-driven algorithms can uncover subtle imaging patterns beyond human perception, while radiomics enables quantitative extraction of high-dimensional features from neuroimaging data. Together, these approaches hold promise for improving diagnostic accuracy, predicting disease progression, and guiding personalized treatment strategies (3). This Research Topic brings together contributions that highlight the latest methodological innovations and clinical applications at the intersection of AI, radiomics, and neurodegenerative disorders, aiming to foster dialogue between researchers, clinicians, and data scientists to accelerate translation into clinical practice.

The Research Topic “*Frontier research on artificial intelligence and radiomics in neurodegenerative diseases*” features 18 studies applying artificial intelligence and radiomics to neurodegenerative disorders: 2 reviews and 16 original research articles. Several of these works highlight the potential of multimodal AI approaches in advancing the diagnosis and management of PD. Zhou, Zhu et al. (“*A predictive model of Parkinsonian brain aging based on brain imaging features*”) demonstrated that specific structural indices such as temporal lobe gyrification and thalamic volume significantly contribute to brain age prediction in PD. Ye et al. (“*Individualized diagnosis of Parkinson's disease based on multivariate magnetic resonance imaging radiomics and clinical indexes*”) showed that integrating radiomic features with clinical data yields superior diagnostic accuracy. Complementing these imaging-based approaches, Yang, Hu et al. (“*Identification of Parkinson's disease using MRI and genetic data from the PPMI cohort: an improved machine learning fusion approach*”) improved early PD diagnosis through MRI–genetic data fusion, while Xu, Xie et al. (“*Non-invasive detection of Parkinson's disease based on speech analysis and interpretable machine learning*”) established speech-based machine learning models that achieved high predictive performance with interpretable features. Together, these studies underscore the promise of AI-driven multimodal strategies—spanning imaging, genetics, and speech—for advancing early, non-invasive, and precise diagnosis of PD.

Advances of AI and radiomics in AD and related cognitive disorders are demonstrated by several studies included in this Research Topic. Xu, Doig et al. (“*Accurate and robust prediction of Amyloid-*β *brain deposition from plasma biomarkers and clinical information using machine learning*”) developed and validated a low-cost, plasma biomarker–based model across the Alzheimer's Disease Neuroimaging Initiative (ADNI) and the Chinese National Twin Network (CNTN) cohorts, achieving high accuracy [area under the receiver operating characteristic curve (AUC) up to 0.95] with only five core features, offering a potential alternative to PET imaging. Yingmei et al. (“*Exploring the changes in functional connectivity of the limbic system in Patients with amnestic mild cognitive impairment treated by acupuncture based on fMRI*”) revealed abnormal limbic connectivity in amnestic mild cognitive impairment and demonstrated that acupuncture could modulate these connections, providing imaging evidence for its therapeutic mechanism. Aresta et al. (“*AI-based staging, causal hypothesis and progression of subjects at risk of Alzheimer's disease: a multicenter study*”) showed that AI tools achieve staging results consistent with expert assessments and accurately align with biomarker-based causal hypotheses in memory clinic workflows. Mao et al. (“*Transcriptional patterns of brain structural abnormalities in CSVD-related cognitive impairment*”) combined neuroimaging and transcriptomics to identify gray matter volume loss in the temporal lobe and thalamus, linking these alterations with 1,580 gene expression profiles and cognitive decline. Zhen et al. (“*Association between Alzheimer's disease pathologic products and age and a pathologic product-based diagnostic model for Alzheimer's disease*”) analyzed large-scale ADNI data, revealing opposite age-related trends of Tau and Aβ levels, and built a high-performance early diagnostic model (AUC = 0.959) using machine learning. Collectively, these studies provide compelling evidence that AI-driven multimodal approaches—ranging from plasma biomarkers and neuroimaging to transcriptomics—hold great promise for the early detection, mechanistic understanding, and precision diagnosis of AD and related disorders.

Beyond Parkinson's and Alzheimer's disease, several studies included here broaden the application of AI and radiomics to other neurological disorders. Fah et al. (“*Comparing machine learning classifier models in discriminating cognitively unimpaired older adults from three clinical cohorts in the Alzheimer's disease spectrum: demonstration analyses in the COMPASS-ND study*”), systematically compared multiple machine learning classifiers and explainable AI techniques, finding Random Forest and Gradient Boosted Trees to be the most robust for near-cohort classification, while SHAP outperformed LIME in interpretability. Huang, Wang et al. (“*Knowledge map of artificial intelligence in neurodegenerative diseases: a decade-long bibliometric and visualization study*”) analyzed 1,921 publications from the past decade, identifying key journals, institutions, and authors, and highlighting future research directions such as multimodal precision diagnosis, mechanistic insights, explainable AI, and technological innovation for specific diseases. Ge et al. (“*Intra- and inter-network connectivity abnormalities associated with surgical outcomes in degenerative cervical myelopathy patients: a resting-state fMRI study*”) integrated static and dynamic resting-state fMRI connectivity to reveal brain network abnormalities with prognostic significance for surgical outcomes. Huang, Li et al. (“*Development and validation of a web-based dynamic nomogram to predict individualized risk of severe carotid artery stenosis based on digital subtraction angiography*”) constructed and validated a non-invasive dynamic prediction model based on six key risk factors in 739 ischemic stroke patients and developed an online tool for individualized clinical decision-making. Xie, Zhang et al. (“*Prediction model for severe autoimmune encephalitis: a tool for risk assessment and individualized treatment guidance*”) built and validated a nomogram based on 207 patients, showing strong discrimination and calibration to guide tailored treatment. Zhou, Li et al. (“*Machine learning-driven prediction model for cuproptosis-related genes in spinal cord injury: construction and experimental validation*”) identified four key cuproptosis-related genes (SLC31A1, DBT, DLST, LIAS) through human data, machine learning, and animal experiments, linking mitochondrial dysfunction and immune response to SCI progression. Finally, Yang, Chen et al. (“*Automatic diagnosis and measurement of intracranial aneurysms using deep learning in MRA raw images*”) developed a fully automated MR 3DUnet model capable of diagnosing and measuring intracranial aneurysms within seconds, demonstrating high accuracy across multiple centers and offering substantial clinical value by improving efficiency and reducing examination time.

The promise of AI-driven approaches for AD is further emphasized by two reviews included in this Research Topic. Peng et al. (“*From pixels to prognosis: radiomics and AI in Alzheimer's disease management*”) discussed the role of PET, MRI, and multimodal imaging in identifying disease-specific biomarkers and predicting progression, emphasizing how AI-enhanced radiomics can improve diagnostic and prognostic precision while also noting challenges in data standardization, model interpretability, and clinical translation. Yuan and Zhao (“*The role of quantitative EEG biomarkers in Alzheimer's disease and mild cognitive impairment: applications and insights*”) underscored the potential of qEEG as a non-invasive, low-cost, and real-time tool for diagnosis and therapeutic monitoring, highlighting biomarkers such as spectral power, connectivity, microstates, and non-linear measures as promising avenues for early detection and precision intervention.

In summary, the Research Topic “*Frontier research on artificial intelligence and radiomics in neurodegenerative diseases*” presents 18 studies that apply machine learning, radiomics, and other artificial intelligence techniques to the study of neurodegenerative diseases. Collectively, these works illustrate how AI-based approaches can advance disease prediction, improve diagnostic accuracy, and support prognosis monitoring. Looking ahead, future research should prioritize clinical translation, validation in large and diverse cohorts, and integration with multimodal data to achieve personalized and precise disease management. These directions will be crucial for harnessing the full potential of AI and radiomics to transform the understanding and treatment of neurodegenerative disorders.
